# Chronic Allopurinol Treatment during the Last Trimester of Pregnancy in Sows: Effects on Low and Normal Birth Weight Offspring

**DOI:** 10.1371/journal.pone.0086396

**Published:** 2014-01-22

**Authors:** Elise T. Gieling, Alexandra Antonides, Johanna Fink-Gremmels, Kim ter Haar, Wikke I. Kuller, Ellen Meijer, Rebecca E. Nordquist, Jacomijn M. Stouten, Elly Zeinstra, Franz Josef van der Staay

**Affiliations:** 1 Emotion & Cognition Group, Department of Farm Animal Health, Faculty of Veterinary Medicine, University Utrecht, Utrecht, The Netherlands; 2 Veterinary Pharmacology, Pharmacotherapy and Toxicology, Faculty of Veterinary Medicine, University Utrecht, Utrecht, The Netherlands; 3 Adaptation Physiology Group of Animal Sciences, Wageningen University, Wageningen, The Netherlands; 4 Pig Health Unit, Department of Farm Animal Health, Faculty of Veterinary Medicine, University Utrecht, Utrecht, The Netherlands; 5 Department of Farm Animal Health, Faculty of Veterinary Medicine, University Utrecht, Utrecht, The Netherlands; 6 Brain Center Rudolf Magnus, Utrecht University, Utrecht, The Netherlands; INRA, France

## Abstract

Low-birth-weight (LBW) children are born with several risk factors for disease, morbidity and neonatal mortality, even if carried to term. Placental insufficiency leading to hypoxemia and reduced nutritional supply is the main cause for LBW. Brain damage and poor neurological outcome can be the consequence. LBW after being carried to term gives better chances for survival, but these children are still at risk for poor health and the development of cognitive impairments. Preventive therapies are not yet available. We studied the risk/efficacy of chronic prenatal treatment with the anti-oxidative drug allopurinol, as putative preventive treatment in piglets. LBW piglets served as a natural model for LBW. A cognitive holeboard test was applied to study the learning and memory abilities of these allopurinol treated piglets after weaning. Preliminary analysis of the plasma concentrations in sows and their piglets suggested that a daily dose of 15 mg.kg^−1^ resulted in effective plasma concentration of allopurinol in piglets. No adverse effects of chronic allopurinol treatment were found on farrowing, birth weight, open field behavior, learning abilities, relative brain, hippocampus and spleen weights. LBW piglets showed increased anxiety levels in an open field test, but cognitive performance was not affected by allopurinol treatment. LBW animals treated with allopurinol showed the largest postnatal compensatory body weight gain. In contrast to a previous study, no differences in learning abilities were found between LBW and normal-birth-weight piglets. This discrepancy might be attributable to experimental differences. Our results indicate that chronic prenatal allopurinol treatment during the third trimester of pregnancy is safe, as no adverse side effects were observed. Compensatory weight gain of treated piglets is a positive indication for the chronic prenatal use of allopurinol in these animals. Further studies are needed to assess the possible preventive effects of allopurinol on brain functions in LBW piglets.

## Introduction

Infants suffering from fetal growth restriction, a pathological decrease in fetal growth rate, are born with a (very) low birth weight ((v)LBW) [1], [2]. LBW children are born with several risk factors for disease, morbidity and neonatal mortality [3]. Though a term born LBW neonate has better prospects for survival than a preterm LBW neonate [4], [5], a child born with LBW is still at risk for developing several health as well as cognitive problems [5]–[12]. The possible causes for LBW vary and may be well-defined (i.e. chromosomal disorders, intra uterine viral infections) [13] or less clearly be attributable to causes such as smoking, obesity, air pollution or placental insufficiency [3], [14]. Placental insufficiency is seen as the most common cause [15] and in general it can be said that a fetus suffering from placental insufficiency adapts to a lack of nutrients or oxygen (hypoxemia) by slowing down growth rate [3], [16].

Various cognitive deficits are associated with being born with (v)LBW in humans. They range from general learning problems [7], [17] to an increased risk for depression [18], schizophrenia [19], anxiety, attention and hyperactivity disorders [20]. Additionally, a reduced brain volume has been found in these children ([11], [21], see also [22]).

Regarding preventive therapies, except for optimizing time of delivery, treatments are not yet available [15]. Pregnant women in developed countries are monitored throughout their pregnancy and receive multiple heart rate and ultrasonographic (with additional Doppler) examinations. This aids pre-partum recognition of the growth restricted fetus by ultrasound technicians and gynecologists [23]. In case of early detection, if a treatment were available to limit the adverse consequences, it could be initiated immediately.

Brain damage, poor neurological outcome, and the mechanisms underlying altered neural development as a consequence of intra uterine growth restriction (IUGR) are not well understood [12], [24]. When oxygen and nutrient supply to the brain is compromised, the fetus attempts to cope with the new situation by protecting its brain by a process called ‘fetal brain sparing’. At the expense of blood trunk supply, more blood is diverted to the brain [25], [26]. However, if compensatory mechanisms are insufficient, fetal distress may ensue and this can have far-reaching consequences extending into adult life [15], [27]. Neuronal cell damage or cell death as a consequence of acute oxygen deprivation of brain tissue has been well studied in many birth asphyxia studies (e.g. [28], [29]). Longer periods of mild oxygen deprivation are expected to occur in IUGR fetuses. These periods can be alternated with periods of re-oxygenation during which oxidative stress may occur, causing additional collateral damage by free radicals produced [14], [28], [30]. Pharmacological intervention with neuroprotective substances, preventing the formation of, or scavenging the free-radicals produced, may improve neurological outcome in these cases. Allopurinol (ALLO) is a candidate anti-oxidative drug with potential neuroprotective properties.

ALLO (1,5-dihydro- 4*H*-pyrazolo[3,4-*d*]pyrimidin-4-one) has been found to reduce free-radical formation in, for example, pig, sheep, and human fetuses [31]–[33]. It is oxidized by the enzyme xanthine oxidase (XO) into the active metabolite oxypurinol (OXY) [34]–[36]. The oxidizing process inhibits the formation of damaging free radicals, and in higher concentrations, ALLO and OXY can also scavenge the free radicals present [36], [37]. ALLO readily crosses the human and pig placenta and does not interfere with the parturition process if administered acutely during parturition [31]. Torrance and colleagues [38] suggested the therapeutic range for neuroprotection to be >2 mg.L^−1^ for ALLO and >4 mg.L^−1^ for the active metabolite OXY. Therefore ALLO seems to be more potent compared to OXY. ALLO is currently being applied in a clinical trial as a therapy preventing damage caused by acute birth asphyxia [39].

The neuroprotective capacities of ALLO have mainly been studied in fetuses and neonates suffering from acute asphyxia during the parturition process. Treatment usually takes place during or after birth. Based on these studies it was suggested that treatment could have a positive effect if 1) ALLO was administered during or as early as possible after asphyxia and 2) the level of asphyxia was not too severe (i.e. that it did not induce irreversible damage) [39]. Under these conditions it is more likely that ALLO treatment is beneficial for the cognitive outcome in LBW children. Non-invasive oral treatment could be started during pregnancy as soon as IUGR has been diagnosed and continue until delivery. The level of hypoxia is expected to be less severe in LBW children compared to children suffering from acute birth asphyxia.

The pig is increasingly used to study neurobehavioral dysfunction because of multiple advantageous characteristics such as its size and brain development [40]. Complementary to these advantages, LBW piglets are a common occurrence in commercial pig rearing [22] due to increasing litter sizes and sow productivity [41]. Therefore, this species could potentially be used as a natural model for IUGR as mechanisms behind growth restriction in pigs and humans are believed to be similar [1]. Poor uteroplacental perfusion is seen as the main cause of growth restriction in pigs [42] and occurs naturally in LBW new-born piglets from large litters [43]. Impaired cognitive performance related to LBW was shown in one of our earlier studies [22] with the cognitive pig holeboard task. Therefore, this putative natural animal model was chosen to study the effects of chronic ALLO treatment.

As only little safety and efficacy data are available about the consequences of prolonged prenatal ALLO treatment in sows and their (IUGR) fetuses [31], we performed three exploratory studies (fully described in Text S2). The first two experiments (S2a and S2b) addressed the pharmacokinetics of oral ALLO treatment in late pregnancy in sows in order to gain more insights in the plasma levels of ALLO and OXY in sows. Subsequently, an exploratory study was performed (see S2c) to assess the feasibility of long term oral treatment and to check for possible adverse effects on placentas and piglets (see Supporting Information S2, experiments S2a and S2b).

Based on the results of these exploratory studies, we performed a study addressing the effects of prolonged ALLO treatment via the sows on LBW and NBW piglets. Several measures were taken from these piglets and from untreated controls, which included piglet characteristics at birth, piglet umbilical cord blood gas values, placental measures (partly derived from experiment S2c, which is fully described in Text S2, behavior in the open field and novel object test for emotionality, performance in a cognitive pig holeboard test for learning and memory and finally body, brain and spleen weight at slaughter (at the age of approximately 5 to 5.5 months). This experiment mainly focused on learning and memory performance of the piglets in the cognitive pig holeboard task. The additional physiological measurements were applied to support and possibly strengthen the behavioral data.

We expected that chronic prenatal ALLO treatment would be safe, i.e. that it would not interfere with the progression of pregnancy or parturition, development of the placenta or growth of the piglets. Further we hypothesized that no cognitive (brain and behavior) or emotional measures of NBW piglets would be influenced by the treatment. LBW piglets treated with ALLO were expected to perform better in the cognitive holeboard test, and show less anxiety in the open field and novel object test compared to LBW controls. Brain, hippocampus and spleen weights were not expected to be influenced by ALLO treatment.

## Materials and Methods

### Ethics Statement

The experimental protocols (DEC numbers 2010.I.06.092 and 2011.I.01.011) were approved by the Animal Experiments Committee of the University Utrecht, The Netherlands. The Animal Experiments Committee based its decision on the EC Directive 86/609/EEC (Directive for the Protection of Vertebrate Animals used for Experimental and other Scientific Purposes). All animal experiments followed the ‘Principles of Laboratory Animal Care’ and refer to the Guidelines for the Care and Use of Mammals in Neuroscience and Behavioral Research (National Research Council 2003). All surgery was performed under ketamine/midazolam anesthesia, and all effort was taken to minimize the number of animals used and their suffering.

Preceding the present study, a series of small experiments was performed (see Table S1 in Text S1, for an overview of all experiments performed). First, we determined the dose and dosing regimen of allopurinol in two exploratory pharmacokinetic studies (experiments S2a and S2b, described in detail in Text S2). Then, a small study assessing the feasibility of treating piglets during the last trimester of pregnancy via oral administration of allopurinol via the sow was performed (experiment S2c, described in detail in Text S2). In this experiment, we checked whether chronic allopurinol treatment had effects on piglet birth weight and the macroscopic appearance and the measures of the placentas. Data collected in this study were combined with data from the present experiment to assess piglet characteristics at birth, placental measures, and their correlations.

#### Animals

Twelve multiparous pregnant sows, a (Terra × Finnish landrace) × Duroc mix, were used. They were from two batches of six sows, with two months between batches (for details, see Table 1).

**Table 1:** Overview of the sows and piglets used.

								LBW and NBW piglets selected for behavioral testing					
Animal	Group	Parity	Litter size plus stillborns	Average litter weight (g)	Number of ♂, ♀ piglets	Plac^1^ (N)	Gas^2^ (N)	LBW: gender, birth weight in g			NBW: gender, birth weight in g)		
**Sow 1**	**Allo**	7	14 (+2)	1303.2	10♂, 4♀	12	2	♂, 755	♂,1040		♂, 1300	♂, 1375	
**Sow 2**	**Allo**	4	13 (+4)	1276.5	7♂, 6♀	12	3	♀, 890	♂, 980		♀, 1400	♀, 1480	
**Sow 3**	**Allo**	2	12 (+2)	1275.8	5♂, 7♀	13	2	♂, 875			♂, 1410		
**Sow 4**	**Control**	6	18 (+1)	1362.7	11♂, 7♀	7	4	♂, 715	♂, 720	♀, 1080	♂, 1660	♀, 1710	♂, 1750
**Sow 5**	**Control**	4	17	1338.8	13♂, 4♀	6	2	♂, 930	♂, 1070	♂, 1085	♂, 1370	♂, 1440	♂, 1340
**Sow 6** *	**Control**	3	10	1855.5	7♂, 3♀	10	2						
**Sow 7**	**Allo**	9	9 (+4)	1607.8	5♂, 4♀	4	5	♂, 1155			♂, 1740		
**Sow 8**	**Allo**	2	18	1439.4	11♂, 7♀	15	10	♀, 860	♂ 1115		♀, 1525	♂, 1700	
**Sow 9**	**Allo**	2	12	1546.7	6♂, 6♀	12	4	♀, 1040	♀ 825		♀, 1665	♀, 1590	
**Sow 10**	**Control**	8	8	1304.4	7♂, 1♀	5	4	♂, 870			♂, 1415	♂, 1460	
**Sow 11** *	**Control**	2	5	2028.0	2♂, 3♀	5	2						
**Sow 12**	**Control**	10	19	0858.9	12♂, 7♀	7	5	♂, 470			♂, 950		

Sows 1 to 6 were from the first batch, sows 7 to 12 were from the second batch. All animals were (Terra × Finnish landrace) × Duroc mix. ALLO sows were treated with 15 mg allopurinol per kg body weight, once daily.

* No LBW piglets were born in these litters, and consequently, no LBW piglets could be selected for the open field test and the holeboard test.

1 Number of piglets for placental measures;

2 Number of piglets for blood gas measures.

#### Housing

Until one week before farrowing, the sows were housed in a group housing system for sows with automatic feeders, straw bedding and *ad libitum* access to water. The sows had free access to an outside area where silage was provided. The ambient inside temperature ranged between 15 and 25°C and light was provided between 07∶00 h and 22∶00 h. Except for one kg of pellets mixed with ALLO, the daily food ration (standard pregnant sow pellets, de Heus, Ede, The Netherlands) was distributed via an automatic feeder.

#### Treatment

Of the twelve pregnant sows (see Table 1), six were treated with ALLO (15 mg.kg^−1^) for 30 days [±2 days depending on the actual farrowing date, starting at day 86 (+1–3 days) of pregnancy] Six untreated sows served as controls. The last ALLO dose was administered on the day of farrowing. The dose of 15 mg.kg^−1^ b.w. was based on a simulation of the plasma-concentration time curve established from two individual sub-experiments in sows (described in Text S2).

### Procedures Around Delivery

#### Sows

One week prior to the expected farrowing date all sows were moved to a conventional farrowing stall (ambient temperature 20–23°C, with floor heating, 30°C, in the piglet area). Food was provided automatically two times a day and access to water was *ad libitum*. The sows returned to the group housing stable after weaning of the piglets.

#### Drug treatment

Based on the results of our exploratory pharmacokinetics studies (see experiment S2a and S2b in Text S2) six sows were treated with allopurinol (15 mg.kg^−1^ b.w.) for 30 days [±2 days depending on the actual farrowing date, starting at day 86 (+1 to 3 days) of pregnancy] and six untreated sows served as controls (see Table 1). ALLO tablets (300 mg, Ratiopharm, The Netherlands) were powdered and mixed with 1 kg of pellets, some honey and water. Animals were observed until all the food was consumed. Sows were weighed weekly to adapt the dose corresponding to their weight gain or loss.

#### Piglets

The piglets were full-term and were delivered vaginally. A series of birth measures were taken (see below), after which the piglets were immediately returned to the sow to drink colostrum. In addition to sow milk, starting at 2–3 days of age artificial milk for piglets (Milkiwean, Trouw Nutrition, The Netherlands) was provided in the pen via a drinking bowl. At 3 days of age all piglets were preventively given an iron injection. When birth diarrhea occurred, all piglets from the affected litter were treated orally with colistine (Enterogel, Virbac Animal Health, Barneveld, The Netherlands) for 3 to 5 days. Crippled piglets before or after weaning were treated with ampicillin (Ampicillan 20%, Alfasan Nederland B.V., Woerden, The Netherlands) for 3 to 5 days and if necessary an analgesic with meloxicam was administered once (Novem 20 mg.ml^−1^, Boehringer Ingelheim Vetmedica GmbH, Ingelheim, Germany). No tail docking, castration or other invasive mutilating procedures were applied in the selected LBW and NBW piglets.

After the piglets had reached 3.5 to four weeks of age the sow was removed and the piglets were weaned. After one to 1.5 additional week(s) in the farrowing pen the selected LBW and NBW piglets were mixed and moved to the two pens in the experimental stable.

The experimental pens (3×5 m) had a concrete floor. A piglet nest (3 m×1 m) could be accessed through rubber flaps. The nest floor was covered with rubber mats, a heat mat (20–30°C, 70 cm×40 cm), sawdust and straw. The pen floor was also covered with straw. Food was provided *ad libitum* during the first 1.5 weeks and twice a day during the rest of the experiment (⅓ before and ⅔ after testing) and was scattered on the pen floor. Water was always available through an automatic drinker. Balls and chewing sticks were provided as extra enrichment materials.

#### Birth measures

Sows were observed constantly starting three days prior to their expected farrowing date. With the onset of labor, at least two experimenters were present to receive the piglet when it was expelled and code the umbilical cord with surgical silk tagged with ‘knots’. These knots were administratively linked to the piglets’ ear tag, which was given after blood sampling. The placental side of the umbilical cord was tagged and the other side was clamped with a kocher. The cord was cut in between at least 7–15 cm away from the piglet. Slightly above the kocher the umbilical cord was cut again and mixed venous/arterial blood (max. 1 ml) was gathered in a 1.5 ml Eppendorf tube. Directly after blood sampling the umbilical cord was disinfected with Betadine and clamped and/or sutured until the bleeding stopped. The blood was directly drawn from the tube into a labeled 1 cc syringe (w/−25 Balanced Heparin, Luer Tip Cap, Westmed, USA) and put on ice. Within 20 minutes the blood sample was cleared from air and analyzed with a portable blood gas analyzer (ABL80 SC80, Radiometer, Zoetermeer, The Netherlands) with a sensor cassette (100/30 Full, no Glu, QC^3^) and pH, _p_CO_2, p_O_2, c_Na^+^, _c_K^+^, _c_Ca^2+^, _c_CL^−^ and Hct were measured.

#### Selection of piglets

After measuring and weighing the piglet returned to the sow and stayed here until weaning. LBW and NBW piglets were determined per litter: the average litter weight and the accompanying standard deviation (SD) were determined per litter. Piglets weighing the mean litter weight minus one SD were classified as LBW. A new litter mean was calculated after exclusion of all LBW piglets in the litter. Animals with a weight closest to this new mean and with the same sex as the LBW animal(s) from the litter were selected as NBW animals. One to three LBW and one to three NBW animals were selected per litter, depending on availability.

#### Placental measures

All placentas that could be gathered from a sow were stored (4°C) and examined within one week to look at any possible adverse effects of ALLO treatment on placental development. Measures included: placenta length (measured along the inside of the placenta from one end to the other); placenta width (measured along the base of the placenta at the broadest point); and placenta circumference (measured by placing a piece of string exactly around the edges of the placenta). All placentas were weighed (10 g accuracy, Breuer Weegtechniek JB-800, Boxtel, The Netherlands). Scaled pictures were taken from above to calculate the surface area using PDF-Xchange Viewer 2.5.

### Behavioral Testing

#### Open field and novel object test

During the fifth week after birth, one week after moving and mixing, an open field and novel object test was performed once with each animal. In a random order but per pen an animal was separated and let into a corridor leading to the test arena. It was let into a small waiting box covered with sawdust as on the test floor. After approximately 30 seconds the animal was released into the open field arena and the door was closed. The arena [250×205 cm (first batch) and 250×150 cm (second batch)] was fenced with wooden and synthetic partitions, at least 1.2 m high. The floor of the open field was covered with sawdust. A radio was playing in the background to mask environmental noise. Video feed from a camera hung above the open field was fed to a monitor, and a transparent sheet placed in front of the monitor divided the open field into 16 equally sized partitions. Behavior was observed from the screen and scored with the custom made software Observe6. Vocalizations were scored independently by another observer, who was not visible to the pig.

The total duration of the test was ten minutes. After five minutes an unknown object (a colorful plastic tambourine) was suddenly lowered by a rope in the middle of the arena and made a noise when touching the floor. Starting from the time point of lowering the object, ‘touching the object’ and ‘looking at the object’ were scored. After ten minutes the animal was led back to its home pen.

#### Holeboard testing

The cognitive pig holeboard apparatus (manufacturer Ossendrijver BV, Achterveld, The Netherlands) consisted of a square arena (530×530 cm) surrounded by a narrow corridor (width 40 cm). Via this corridor, four guillotine entry doors could be reached to access the arena. In the arena a 4×4 matrix of food bowls was placed. Rewards in a bowl could be found by lifting the balls on top with the snout. For a detailed description of the apparatus see Gieling et al. [22]. The holeboard apparatus could be accessed via a group waiting pen. Animals were always tested individually in the apparatus and a radio played continuously to mask background noises.

Each food bowl was equipped with a magnet sensor and every ball with a magnet. A visit was counted when a pig lifted the ball on top of the food bowl with its snout. A signal between a magnet sensor placed under the false bottom and a magnet in the ball was now disconnected. Registration of the hole visits and automatic termination of the trial was done by an interface (LabJack Data Acquisition Device, LabJack Corporation, USA) connected to a laptop running custom made software (Blinq Systems, Delft, The Netherlands). Raw data were stored as a text file, and in parallel, pre-processed data were output to a Microsoft Excel file.

#### Habituation

Five weeks after birth habituation started. An experimenter sat in the pen and touched the animals gently when possible. M&M chocolates and some corn cob mix were given to attract attention of the piglets and to get them used to the rewards. Piglets were habituated to the corridor leading to the holeboard waiting area and the waiting area itself (a pen with an automatic drinker and straw bedding). As a group they were led into the holeboard using different entry doors. All food bowls were rewarded with M&M chocolates. After three group sessions the group was split in two and habituation to the holeboard was repeated, using only M&M’s as rewards. Finally, the animals were tested in groups of two individuals (approx. 4 times) and alone (approx. 4 times). The entire habituation period lasted 13 working days.

**Figure 1:**
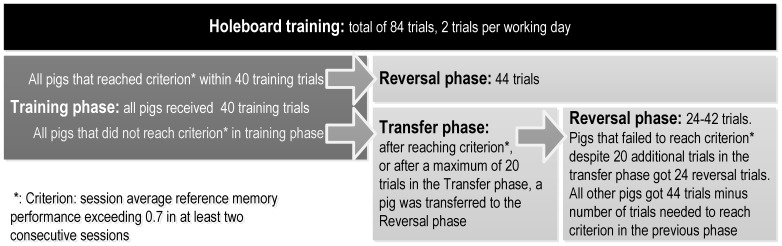
Timeline of holeboard training.

An animal was defined ready for testing when it was able to stay in the holeboard for at least 60 seconds while searching for rewards under the balls. For the first training trial, all animals entered the holeboard through door nr. 1. On all following trials, the entry door was assigned randomly by the software. A specific door was never assigned to an animal more than twice in a row. Every trial lasted until the 4^th^ reward was found or ten minutes had elapsed, whichever event occurred first. Every animal was assigned two successive trials a day (one session) in a random order. The training phase in which every animal was assigned one specific configuration of rewarded bowls (4 out of 16) lasted for at least 40 trials per animal. Four different configurations were used (the configuration as depicted in Fig. 4E in [44]) and three variants turned 90°, 180° and 270°). A performance criterion (session average reference memory performance >0.7 for at least two consecutive sessions) was set before an animal was allowed to switch from the training configuration (training phase) to a new one (reversal). All animals were switched to the reversal if not reaching criterion after a maximum number of 60 training trials (transfer phase). In total all animals were trained for an equal number of trials (84), although the number of training and reversal trials differed per animal (outlined in Figure 1).

Holeboard measures are memory, motivation or strategy related. (Re)visits to rewarded bowls, (re)visits to unrewarded bowls and total trial duration were measured. For scoring revisits two specific rules were applied: a revisit only was counted as such if at least ten seconds had elapsed between the previous visit to the same bowl or when another bowl was visited in between.

Several measures were derived from the raw data:

**Working memory ratio (WM):** (number of rewarded visits)/(number of visits and revisits to the rewarded set of bowls). WM is seen as a short term memory measure, reflecting the ability of the animals to avoid revisiting baited bowls [45].

**Reference memory ratio (RM):** (number of visits and revisits to the rewarded set of bowls)/(number of visits and revisits to all bowls). RM is seen as a long term memory measure and is an index for the ability of an animal to discriminate between baited and unbaited holes [45].

Ratio measures were used as these are less biased by incomplete trials, in which the animal does not collect all rewards [44].

**Trial duration (TD):** the time elapsed between entering the holeboard and finding the last reward with a maximum of 600 seconds if not all rewards were found.

**Inter visit interval (IVI):** the average time between visits to bowls (s).

**Trials to criterion (TC):** The number of trials an animal needs (with 40 as a minimum) till reaching criterion to start with reversal training.

**Response flexibility (RF):** The delta of performance (WM, RM, TD and IVI) of the last trial block of the first configuration, compared with the performance of the first trial block of the second configuration (reversal). The larger the difference (delta), the more difficulty an animal showed to adapt to a new situation.

**Choice** **Correspondence (CC):** Visiting order of first visits to the four rewarded bowls. This measure can give insight in the strategy an animal applies to solve the task. An animal could repeatedly follow the same strategy or alter it depending on the situation (e.g. entry door) to maximize gain [44]. CC is calculated according to the rules described by van der Staay et al [44]. With no strategy animals would score an average of 1.72 and a higher performance indicates use of a searching strategy. When the exact same visiting order would be applied repeatedly, the maximum score of 4 would be reached.

**Errors per reward (EpR):** This newly defined measure analyses the number of errors [incorrect (re)visits] an animal makes before finding each of the four rewards in a trial. The errors are counted per reward found (before finding reward 1, between finding reward 1 and 2, 2 and 3, and 3 and 4) i.e. four successive values are determined that are not accumulated over rewards. These four values are compared with each other per block of trials (four trials per block) and per body weight group to analyze whether the EpR increase across successive rewards. This measure can be used in a descriptive way to interpret the difficulty level of the test per treatment group. If the number of errors increases when later rewards are still to be found, memory load is probably increasing, or executive-attention to fulfill the task correctly is declining or eventually lost. An executive-attention component deficit is suggested to be one of the causes of cognitive impairment in IUGR children [46].

### Data Analyses

#### Placental measures

The large number of placentas which could not be traced back to a specific piglet precluded an analysis of the effects of birth weight. Consequently, this factor was not included in the analysis of the placenta measures. Using SPSS 16.0 for Windows placenta measures were analyzed using a linear mixed model with ‘treatment’ as a fixed and ‘sow’ as a random factor. ‘Litter size’ was used as covariate as the litter size is a major determinant of the weight of the piglet in a litter, with larger litters having smaller piglets [47].

Placenta’s collected in an experiment fully described in Text S2 (experiment S2c) and the placentas collected in the present experiment were combined for analysis. Pearson correlation coefficients and Spearman’s correlation coefficients were calculated to check for correlations between placenta variables. Piglet birth weight was included in the analyses to search for correlations between birth weight and placental variables. Data were analyzed with SPSS 16.0 and SAS 9.2.

#### Piglet measures and blood gas data

Using SPSS 16.0 for Windows, piglet measures and blood gas data were analyzed using a linear mixed model for the umbilical cord blood gas values and piglet measures data. ‘Treatment’ was set as a fixed factor and ‘sow’ as a random factor. ‘Litter size’ was used as covariate.

#### Open field and novel object data

Open field measures include line crossings (LC, an activity measure), the number of vocalizations (V), the number of defecations (D) (all during ten minutes), looking at the novel object (LNO), and touching the novel object (TNO) (both measures during the second 5 min of observations).

Using SPSS 16.0 for Windows, the data were analyzed using a linear mixed model with ‘treatment’ as a fixed factor, ‘sow’ as a random factor and ‘litter size’ as a covariate. Birth weight class (LBW or NBW) was added as well as the interaction ‘treatment by birth weight class’ as fixed factors. Each variable was checked for normality by plotting parameter estimates against parameter residuals in a Q-Q and scatter plot. Significance level was fixed at ≤0.05.

#### Holeboard data

The animals were trained for two consecutive trials a day (one session). For each measure, block mean values of four trials (two sessions) were calculated (methods adapted from [22], [45]). The data was analyzed with SAS 9.2. NBW and LBW piglet data was averaged per sow (treated or control) and the repeated measures data (blocks of four trials each, or doors) of each sow were used in the analysis. Therefore, for each variable two measures per trial block for each sow were tested in a General Linear Model for Repeated Measures with trial blocks or doors as second repeated measures factor. Every variable was checked for normality with a Shapiro-Wilk test for normality. Significance level was fixed at ≤0.05.

#### Body, brain and spleen weights

The data was analyzed with SAS 9.2. NBW and LBW piglet data was averaged per sow (treated or control). To calculate relative weights, the weight of the brain or spleen was divided by the final body weight of the animal. The two variables for each sow were tested in a General Linear Model for Repeated Measures with birth weight as repeated measures factor. Every variable was checked for normality with a Shapiro-Wilk test for normality. Significance level was fixed at ≤0.05.

## Results

### Blood Gas Measures

A maximum of 2–10 umbilical cord mixed blood samples per sow could be collected from the piglets. It was not always possible to draw blood from an umbilical cord vein after the cord was cut or broken. The pH values of the samples did not include any values below 7.0. The pCO2 values did not exceed 100 mm Hg, although two samples had a very low pCO2 concentration (<25 mm Hg). No effect of allopurinol treatment was found on any of the blood gas parameters. The data is shown in Table 2.

**Table 2:** Effects of Allopurinol treatment on piglet birth measures.

Measure					ALLO treated			Controls		
		F	DF	P <	Mean	SEM	N	Mean	SEM	N
Placenta	Length (cm)	**4.886**	1,14.335	**0.044**	63.86	1.64	74	74.95	2.60	51
	Width (cm)	0.249	1,13.398	0.626	15.67	0.26	70	15.96	0.28	48
	Circumference (cm)	2.557	1,15.216	0.130	147.47	3.47	74	167.25	5.52	51
	Surface area (cm^2^)	0.320	1,13.888	0.581	583.64	30.73	69	624.97	37.32	45
	Weight (g)	0.200		0.663	0.273	0.01	74	0.266	0.01	51
Piglets	Birth weight (g)	3.044	1,12.463	0.106	1470.97	31.61	105	1255.08	37.55	103
	Full length (cm)	**6.347**	1,12.421	**0.026**	37.296	0.34	93	33.203	0.49	72
	Snout length (cm)	0.081	1,12.687	0.387	12.350	0.18	94	11.232	0.18	74
	Ponderal index	1.479	1,11.907	0.247	27.693	0.46	93	33.502	1.39	72
Blood gases	pH	1.603	1,8.686	0.238	7.377	0.02	26	7.420	0.02	19
	pCO2	0.008	1,8.229	0.930	47.35	1.80	26	45.58	2.15	19
	pO2	1.217	1,3.540	0.339	39.62	6.50	26	52.37	8.98	19
	Hct	0.315	1,8.910	0.588	25.56	0.65	26	26.67	1.86	19

Degrees of freedom, F-values, associated probabilities, mean, SEMs and Ns op the ALLO-treated and untreated control piglets are listed.

Differences in birth measures between piglets from the six sows treated with allopurinol and four control sows. Note that the data of two sows of the control condition (no ALLO treatment) were not used because they did not give birth to LBW piglets (see Table 1 for details). Full body length (cm) = snout to tail base; snout length (cm) = snout to end of skull; ponderal index = weight/length3.

### Piglet Birth Measures

During delivery no complications occurred. The lengths of parturition fell within the normal range and all placentas were released naturally.

Full body length in ALLO treated piglets exceeded that of the control piglets. No treatment effects were found for snout length, birth weight or ponderal index (Table 2). Piglets selected as LBW and NBW animals for behavioral testing ranged at birth between 470 g and 1155 g (LBW: ALLO average weight 956 g; CONT average weight 867 g) and 950 g and 1750 g (NBW: ALLO average weight 1519 g; CONT average weight 1455 g).

### Placental Measures (Data from Experiment S2c in Text S2), and from the Present Experiment)

A total of 125 placentas derived from 17 sows (from the 5 sows of exp. S2c, see Text S2, and from the12 sows of the present study) were collected (see Table 2) during three data collection periods (piglets of the 5 sows of experiment S2c in Text S2, that were delivered by Caesarean section; and piglets of the 12 sows of the present experiment, that were vaginally delivered). From these placentas, 57 could be linked to specific piglets. From sow 12 of the present study no piglets could be linked to their placentas (see Table 1).

Placenta length was found to be shorter in ALLO treated piglets than in control piglets. No other effects of treatment were found for placenta width, circumference or surface area (see Table 2).For the following correlation analysis, data from exp. S2c, reported in Text S2, and from the present experiment were combined.

Pearson product moment correlation coefficients between the placental measures and of placental measures with birth weight are shown in Table 3. Placenta length was correlated with circumference (*r_PM_ = *0.945, *P* = 0.000), surface area (*r_PM_* = 0.597, *P* = 0.015) and placenta weight (*r_PM_* = 0.502, *P* = 0.040). Placenta width was only correlated with weight (*r_PM_* = −0.614, *P* = 0.009). Placenta circumference correlated with length (*r_PM_* = 0.945, *P* = 0.00) and surface area (*r_PM_* = 0.543, *P* = 0.024). Placenta surface area correlated with length (*r_PM_* = 0.597, *P* = 0.015), and weight (*r_PM_* = 0.700, *P* = 0.002). Placenta weight correlated with length (*r_PM_* = 0.502, *P* = 0.040), width (*r_PM_* = −0.614, *P* = 0.009), and surface area (*r_PM_* = 0.700, *P* = 0.002). Finally, piglet birth weight correlated with placenta circumference (*r_PM_* = 0.642, *P* = 0.007) but not with any other measure.

**Table 3:** Correlation between placental measures and birth weight.

		Placenta width	Placenta circumference	Placentasurface area	Placentaweight	Pigletbirth weight
Placenta length	*r_PM_*	−0.176	**0.945**	**0.579**	0.502	0.488
	(N) *P* <	(17) 0.500	(17) 0.000	(17) 0.015	(17) 0.040	(16) *0.055*
Placenta width	*r_PM_*		−0.027	−0.270	**−0.614**	0.188
	(N) *P* <		(17) 0.917	(17) 0.295	(17) 0.009	(16) 0.486
Placenta circumference	*r_PM_*			**0.543**	0.361	**0.642**
	(N) *P* <			(17) 0.024	(17) 0.154	(16) 0.007
Placenta surface area	*r_PM_*				**0.700**	0.180
	(N) *P* <				(17) 0.002	(16) 0.504
Placenta weight	*r_PM_*					−0.045
	(N) *P* <					(16) 0.867

Pearson’s product moment correlation coefficients (rPM), the associated probabilities (two tailed), and the number of measurements (Ns) are listed. Correlation coefficients with associated probabilities <0.05 are printed bold, whereas coefficients with 0.1 >*P* >0.05 are printed in italics.

### Open Field and Novel Object

In total 37 piglets were tested in the open field test with the number of piglets tested per sow ranging between 1 and 3 per birth weight group. The only effect found was that LBW piglets vocalized more than NBW animals [*F*_1,7_ = 4.895, *P* = 0.036; LBW (mean ± SEM): 327.61±56.01), NBW: 238.05±54.61)]. Neither effects of treatment nor treatment by birth weight interactions were found.

### Holeboard

Results for Working memory (WM), Reference memory (RM), Trial duration (TD), Inter-visit-interval (IVI), Trials to criterion (TC), Choice correspondence (CC), and Response flexibility (RF) are listed in Table 4.

**Table 4:** Performance of ALLO-treated and untreated LBW and NBW pigs in the cognitive pig holeboard task.

			Treatment(TM)			Blocks(Bs)			Birthweight (BW)			BW×TM			BW×Bs			Bs×TM			BW×Bs×TM		
	Measure	Phase	*F*	DF	*P* <	*F*	DF	*P* <	*F*	DF	*P* <	*F*	DF	*P* <	*F*	DF	*P* <	*F*	DF	*P* <	*F*	DF	*P* <
Learning	Trial Duration	training	0.08	1,8	0.788	18.59	9,72	**<0.001**	0.04	1,8	0.855	0.62	1,8	0.454	1.62	9,72	0.126	0.9	9,72	0.530	1.16	9,72	0.334
		reversal	0.20	1,8	0.670	90.13	5,4	**<0.001**	0.07	1,8	0.803	1.23	1,8	0.313	0.18	5,40	0.967	1.23	5,40	0.313	0.27	5,40	0.925
	Working Memory	training	0.04	1,8	0.841	8.94	9,72	**<0.001**	0.39	1,8	0.550	1.10	1,8	0.325	0.74	9,72	0.667	1.03	9,72	0.423	1.55	9,72	0.147
		reversal	0.32	1,8	0.584	26.63	5,4	**<0.001**	0.09	1,8	0.773	0.04	1,8	0.847	0.58	5,40	0.717	0.09	5,40	0.992	1.11	5,40	0.368
	Reference Memory	training	0.42	1,8	0.537	63.21	9,72	**<0.001**	1.03	1,8	0.339	0.35	1,8	0.569	0.67	9,72	0.736	0.33	9,72	0.961	0.52	9,72	0.855
		reversal	0.46	1,8	0.519	61.16	5,4	**<0.001**	0.02	1,8	0.886	0.27	1,8	0.618	0.27	5,40	0.926	0.54	5,40	0.741	1.74	5,40	0.223
	Inter Visit Interval	training	0.12	1,8	0.704	7.06	9,72	**<0.001**	0.04	1,8	0.855	0.12	1,8	0.739	1.15	9,72	0.337	0.65	9,72	0.750	0.52	9,72	0.859
		reversal	0.05	1,8	0.822	32.08	5,4	**<0.001**	0.07	1,8	0.799	0.84	1,8	0.386	0.21	5,40	0.958	3.28	5,40	**0.014**	0.29	5,40	0.914
	Trials to Criterion	training	0.30	1,8	0.598	*n/a*	*n/a*	*n/a*	2.90	1,8	0.127	1.10	1,8	0.325									
Response flexibility	Trial Duration	transition	0.85	1,8	0.384				0.07	1,8	0.795	0.34	1,8	0.575									
	Working Memory	transition	0.33	1,8	0.579				0.69	1,8	0.431	1.22	1,8	0.301									
	Reference Memory	transition	1.18	1,8	0.309				2.57	1,8	0.148	0.09	1,8	0.774									
	Inter Visit Interval	transition	1.52	1,8	0.254				0.15	1,8	0.706	0.63	1,8	0.449									
Strategies	Block differences	training	0.14	1,7	0.717	0.56	9,63	0.821	0.17	1,7	0.689	1.38	1,7	0.279	1.02	9,63	0.432	0.67	9,63	0.729	1.30	9,63	0.255
	Door differences*	training	0.08	1,8	0.781	1.29	3,24	0.301	0.03	1,8	0.865	0.38	1,8	0.554	0.71	3,24	0.558	0.97	3,24	0.423	1.16	3,24	0.346

The transition from the originally learned pattern of baited holes to a new pattern (reversal) was taken as index for response flexibility. Strategies are reflected by the measure choice correspondence and have been analyzed across block of trials without considering through which pigs entered the holeboard arena (block differences), and also per door. n/a: not applicable.

Differences between 4 treatment groups: Performance of LBW and NBW piglets born from allopurinol treated sows (n = 6) and controls (n = 4).

**Figure 2:**
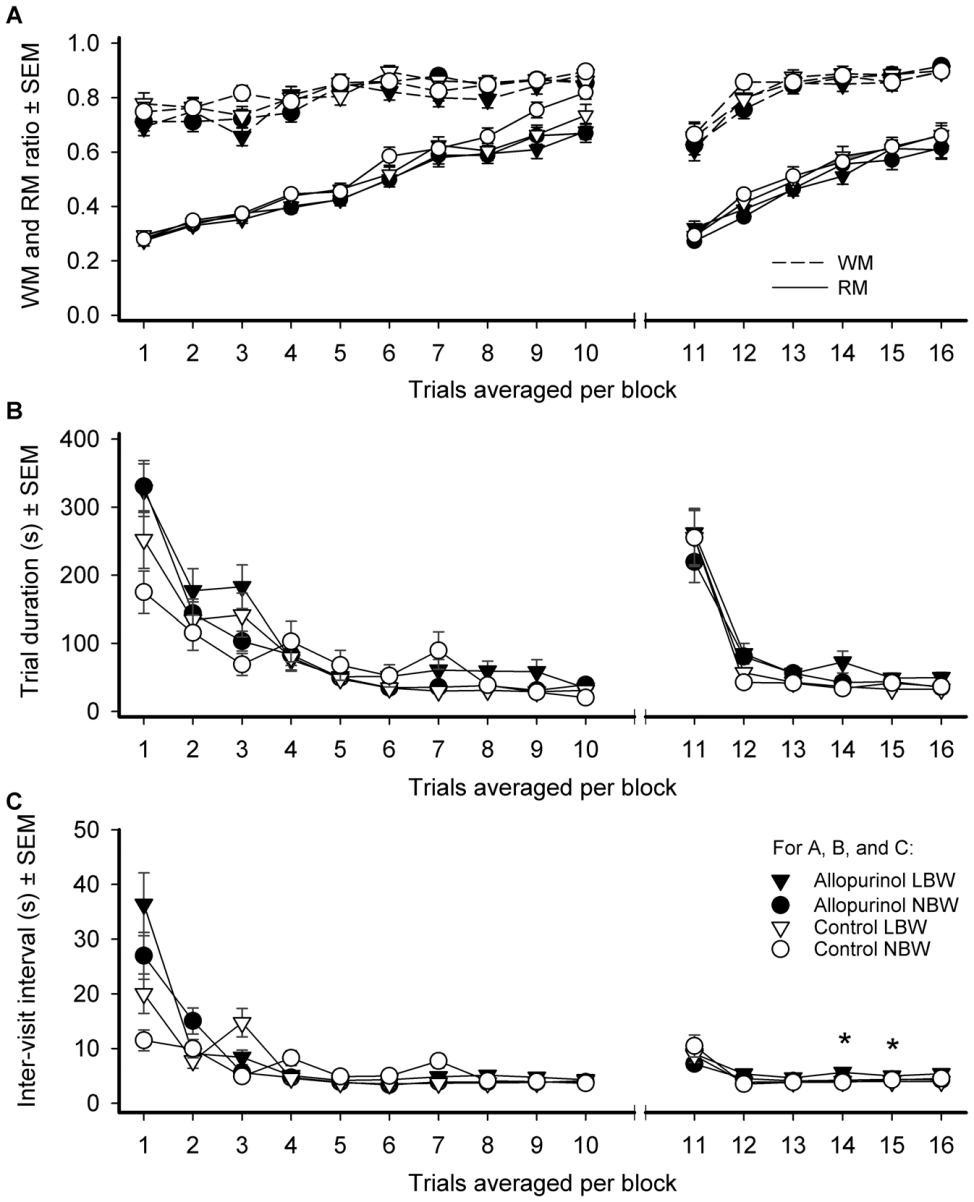
Behavior of four different treatment groups in a spatial holeboard task. *Groups*: low birth weight (LBW) and normal birth weight (NBW) piglets, prenatally treated with allopurinol and untreated controls. Means and SEM for the ten trial blocks of the training phase (1–10) and six trial blocks of the reversal phase (11–16) are shown for (A) WM (stippled lines) and RM (solid lines), (B) trial duration and (C) IVI.

Training improved performance (block effect for WM, RM, TD, and IVI both for the training and the reversal phase; see Figure 2). An interaction effect between blocks and treatment was found for the measure IVI during the reversal phase (*F*_5,40_ = 3.28, *P*<0.014). ALLO treated piglets had a longer IVI during blocks 13 and 14 of reversal training (contrast variables block 13: *F*_1,8_ = 5.64, *P*<0.001; block 14: *F*_1,8_ = 8.28, *P*<0.021). No other treatment, birth weight or treatment by birth weight group interaction effect was found for the training or reversal phase for the measures WM, RM, TD, and IVI. Comparing the RF and TC of the four groups, no effects of birth weight or ALLO treatment were found.

#### Choice Correspondence

Comparing the visiting order of rewarded visits per block of four trials for the first ten blocks of training, none of the four groups significantly changed their strategy over the blocks (*F*_9,63_ = 0.56, *P* = 0.8211) and the slopes showed no differences between groups (data not shown). The average CC calculated per door over 40 training trials did not show different levels of food searching strategies per door, neither did treatment or BW group influence CC. Delta’s of mean CC score per door per BW group minus performance at chance level (1.72) showed that strategy performance for all doors was significantly above random performance level (LBW group: door 1, *t*_9_ = 2.86, *P* = 0.0.019; door 2, *t*_9_ = 5.03, *P* = 0.001; door 3, *t*_9_ = 3.74, *P* = 0.005; door 4, *t*_9_ = 4.37, *P* = 0.002; NBW group: door 1, *t*_9_ = 3.24, *P* = 0.010; door 2, *t*_9_ = 4.64, *P* = 0.001; door 3, *t*_9_ = 4.18, *P* = 0.002; door 4, *t*_9_ = 4.08, *P* = 0.003).

#### Errors per reward (EpR)

**Figure 3:**
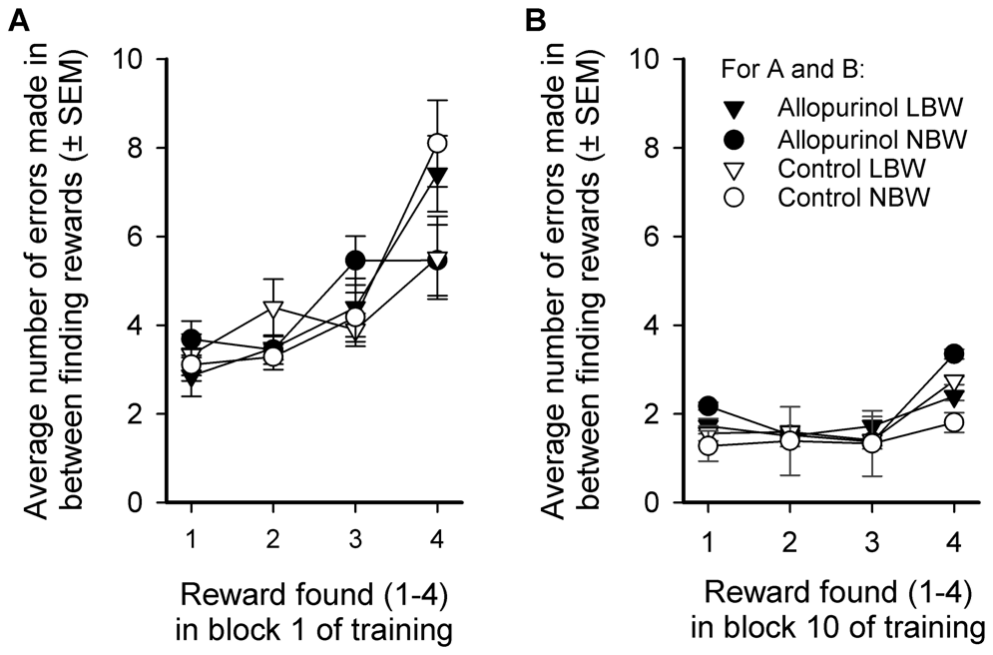
Average number of errors* made per birth weight and treatment group between finding rewarded bowls. **X-axis:** 1: before locating the 1^st^ reward, 2: between locating reward 1–2, 3: between locating reward 2–3, 4: between locating reward 3–4. Groups: low birth weight (LBW) and normal birth weight (NBW) piglets, prenatally treated with allopurinol and untreated controls. Means and SEM for the 1^st^ (panel A) and 10th trial block (panel B) of the training phase are shown. *Error = visiting an unrewarded or previously rewarded bowl. **Trial block = 2 sessions of 2 consecutive trials (i.e. 4 trials in total).

During the first and the 10^th^ block of training, the number of errors made before finding the first bowl containing a reward and between finding the following rewards increased in order of the rewards obtained (see Figure 3A, block 1: *F*_1,7_ = 12.53, *P*<0.001; Figure 3B, block 10: *F*_3,24_ = 6.79, *P*<0.002). ALLO treatment or birth weight or their interaction did not affect EpR.

### Body, Brain, Hippocampal, and Spleen Weight

**Figure 4:**
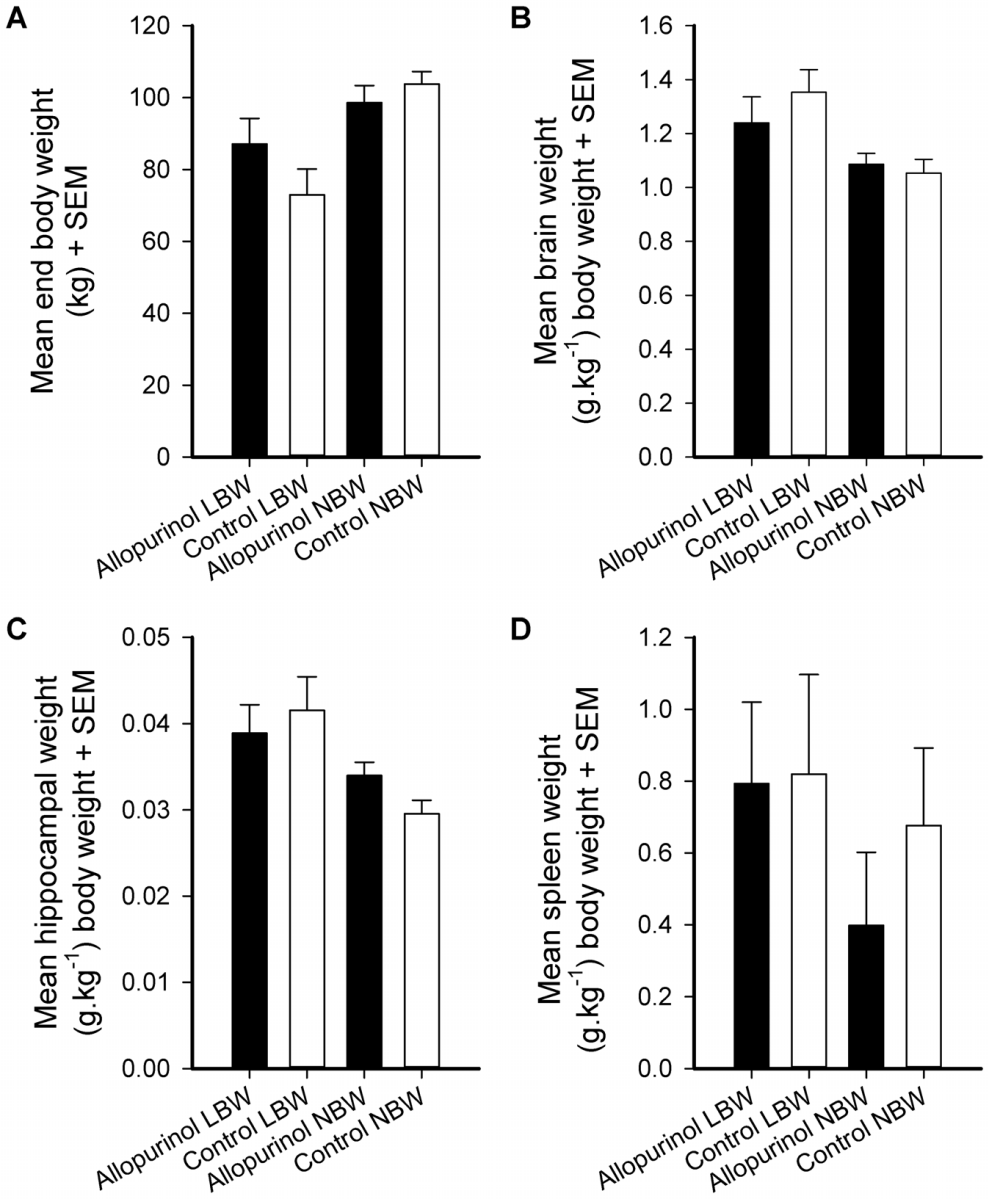
Absolute body and relative organ weights of low (LBW) and normal birth weight (NBW) pigs derived from allopurinol treated and control sows at the age of 5 and 5.5 months. Panel A: body weight, panels B–D: relative organ weights. Ratios are calculated by dividing the organ weight through the end body weight per animal.

No overall effect of ALLO treatment on final (slaughter) weight of the pigs (age between 5 and 5.5 months) was found. There was a marginal interaction between birth weight and treatment (see Tables 5 and 6). LBW control animals seemed to have a lower body weight compared to the LBW animals treated with ALLO. This didn’t seem to be the case for the NBW groups (see Figure 4A). Overall, LBW animals had a lower slaughter weight than NBW animals (*F*_1,8_ = 5.20, *P*<0.005). Brain weights (see Figure 4B) and hippocampus weights (see Figure 4C) did not differ between the ALLO treated animals and the controls. Spleen weight, however, was marginally lower in the ALLO treated animals (*F*_1,8_ = 4.23, *P*<0.007, see Figure 4D).

**Table 5:** Effect of birth weight, ALLO treatment and their interaction on slaughter weight, and relative hippocampal and spleen weight (for means and SEMs see Table 6).

	Treatment (TM)			Birth weight (BW)			BW×TM		
Measure	*F*	DF	*P* <	*F*	DF	*P* <	*F*	DF	*P* <
End body weight	0.27	1,8	0.616	5.20	1,8	**0.005**	3.92	1,8	*0.083*
Mean brain weight	0.19	1,8	0.676	6.15	1,8	**0.038**	0.08	1,8	0.791
Mean hippocampal weight	0.65	1,8	0.443	6.87	1,8	**0.031**	0.33	1,8	0.581
Mean spleen weight	4.23	1,8	*0.074*	0.22	1,8	0.655	0.14	1,8	0.715

**Table 6:** Absolute and relative weights per birth weight by treatment group.

	Absolute weights						Relative weights (organ weight/end body weight)					
	Group	Mean	SEM	Group	Mean	SEM	Group	Mean	SEM	Group	Mean	SEM
End body weight (kg)	LBW ALLO	87.10	7.10	NBW ALLO	98.60	4.75						
	LBW CONT	73.00	7.15	NBW CONT	103.78	3.47						
Brain (g)	LBW ALLO	102.00	1.51	LBW ALLO	105.60	2.51	LBW ALLO	1.24	0.10	LBW ALLO	1.09	0.04
	LBW CONT	99.00	4.63	LBW CONT	108.11	2.85	LBW CONT	1.35	0.08	LBW CONT	1.05	0.05
Hippocampus (g)	LBW ALLO	3.18	0.06	LBW ALLO	3.30	0.09	LBW ALLO	0.039	0.0033	LBW ALLO	0.034	0.0015
	LBW CONT	2.86	0.27	LBW CONT	3.03	0.10	LBW CONT	0.042	0.0039	LBW CONT	0.030	0.0015
Spleen (g)	LBW ALLO	118.00	9.46	LBW ALLO	121.30	6.10	LBW ALLO	0.79	0.23	LBW ALLO	0.34	0.20
	LBW CONT	104.29	11.49	LBW CONT	131.78	6.98	LBW CONT	0.82	0.28	LBW CONT	0.68	0.22

The means and standard errors of the mean (SEM) are listed.

Brain and hippocampus ratio measures were influenced by birth weight but not by treatment. In absolute terms the brains of LBW pigs weighed significantly less than the brains of NBW pigs (analysis not shown, mean brain weight LBW animals 100.5 g; mean brain weight NBW animals 106.86 g). The relative brain and hippocampus weights, i.e. the weights expressed per kg body weight, were found to be higher in the LBW than the NBW animals (*F*_1,8_ = 6.15, *P*<0.005 and *F*_1,8_ = 6.87, *P*<0.031 respectively).

## Discussion

The final aim of the present series of experiments was to assess the safety and efficacy of chronic prenatal oral treatment with ALLO of the sow on her piglets postnatally. Mainly parameters related to cognition were measured but emotional reactivity and growth were also taken into account. The progress of pregnancy and delivery including the placenta were registered for safety reasons. All sows were observed starting before the onset of parturition until the end of the weaning period. No adverse effects of chronic allopurinol treatment were found on farrowing, birth weight, open field behavior, learning abilities, relative brain, hippocampus or spleen weights. LBW piglets showed increased anxiety levels in an open field test for emotional reactivity, but neither piglet working nor reference memory was affected by allopurinol treatment. LBW animals treated with ALLO showed the largest postnatal compensatory body weight gain. During delivery no complications occurred. The lengths of parturition fell within the normal range and all placentas were released naturally.

### Blood Gas Levels

Blood gases were measured immediately after birth to identify piglets that may have suffered from acute birth asphyxia. Allopurinol did not influence blood gas values of neonatal piglets prenatally treated from day 86 (+1–3 days) of gestation including the day of delivery. None of the sampled piglets seemed to have suffered from acute asphyxia as no pH levels below 7.0 or pCO_2_ levels above100 mDobberkem HG were measured [48]–[50], though these levels are generally based on venous or arterial blood samples rather than the mixed samples drawn in the present experiment. Variation between pH and pCO_2_ levels in mixed, venous or arterial blood of piglets is unknown. In total 35% of the behaviorally tested piglets were sampled for blood gas values. As blood samples couldn’t be drawn from all piglets, this might implicate that an unwanted bias selected piglets that were easy to sample. Umbilical cords from specific sows seemed to break much easier compared to cords of other sows, hindering blood sampling. Blood samples were drawn as soon as possible after delivery and cutting the umbilical cord, but gas exchange through breathing could not be prevented. This could have influenced the results [50]. Some analyses failed due to excessive air in the samples. Possibly, the level of oxidative stress determined by blood gas parameters may in the future be substituted or complemented by evaluating (anti-)oxidative parameters of placental tissue or maternal plasma [14].

### Placental Features and Body Measures

To assure that chronic ALLO treatment had no effects on general placental features, basic measures were taken from placentas of treated and control piglets. Most placental features were unaffected by prolonged ALLO treatment, except that placentas derived from treated piglets were found to be shorter. This contrasts with our finding that body length is longer in the ALLO treated piglets. According to Wilson [51] placental size is inversely correlated with its efficiency, i.e. smaller placentas seem to be relatively more efficient. As the ALLO treated piglets are found to be taller but not heavier compared to controls, placenta length does not seem to be a factor of biological relevance for the health and viability of ALLO treated piglets.

### Open Field and Novel Object Test

To assess the effects of ALLO treatment on the anxiety level of the piglets, an open field and novel object test (combined) was performed just after weaning. Emotional reactivity of ALLO treated piglets in the open field test did not seem to differ from that of the controls in the LBW or the NBW group. However, LBW piglets vocalized more than NBW piglets. Increased vocalizations in piglets are shown to be correlated with unpleasant or painful situations [52] and social isolation [53], all known to be stressful and anxiety inducing events. Epidemiological studies in (v)LBW children showed anxiety to be increased [20], which suggests that increased anxiety related to LBW is shared amongst humans and pigs.

Stress-reducing drugs as azaperone are found to decrease the number of vocalizations in piglets when subjected to a stressful environment [53]. Piglets born from cortisol treated sows, a prenatal stress model, were found to vocalize more compared to controls in a novel environment test in a study by Kranendonk et al [54]. Our finding that LBW piglets vocalize more in the open field test compared to NBW sibling corroborates findings of Weary et al. [55], who found that isolated LBW piglets vocalized more than NBW piglets. Chronic ALLO treatment did not affect the increased anxiety levels in LBW piglets in any direction (mean frequency ± SEM; LBW ALLO: 318.80±49.92; LBW CONT: 338.63±30.29).

### Holeboard

The measures WM and RM clearly showed that all four groups were well able to learn both the initial configuration and a reversal of the holeboard task. These results are in line with earlier pig holeboard studies [22], [45], [56]. However, one of our earlier studies [22] showed LBW animals to have more difficulty with the transition from one learned configuration to a new one (reversal), compared to NBW siblings. This difference in WM performance was not found in the current experiment. A methodological difference between the studies was the moment at which the reversal was commenced. In the experiment by Gieling et al. [22] all pigs started the first reversal after 26 trials. The current experiment applied a RM performance criterion before switching to the reversal and a minimal number of 40 trials during the acquisition phase of the first configuration. Especially the response flexibility (see Figure 2) of WM, RM and TD (calculated as difference score between the end of training on the 1^st^ configuration of baited holes minus performance at the start of reversal learning) clearly shows that if all animals reached a minimal performance level before they start learning a new configuration, they switch easier to the new configuration of baited holes. The general rules of the task (which are RM related [57]) might have been stored better after a longer training period, which makes switching easier. Another difference was that trials were presented once a day as a set of two massed trials. In the previous study two trials a day were given but they were spaced over the day. To be able to conclude whether WM and RM performance under these different conditions truly differ, we analyzed performance of the untreated LBW and NBW animals and compared it with the results of Gieling et al. [22] after 26 training trials. WM and RM performance under the two given circumstances was found to be very similar. In the present study the sow was the unit of treatment (and analysis) and not the individual piglets. This causes a loss of statistical power and affects the sensitivity of tracing possible subtle behavioral differences between groups.

Human studies comparing cognitive performance of LBW children with healthy controls differ substantially for their setup. Not all of them found LBW (but term) born children to be affected by prenatal growth restriction later in life [5]. Altogether we could speculate that not all LBW piglets are clearly cognitively affected by growth restriction. A clear discrepancy between the present pig study and human studies is that our piglets were kept under conventional farm circumstances which are not optimal for survival of the piglets which are most severely affected by growth restriction. As survival rates of the most affected LBW children is improving in the western world [58], optimally this high level of neonatal care should be imitated in the translational pig studies to ensure inclusion of the most affected animals and avoid a bias through loss of the less viable animals.

Except for a treatment by block interaction effect on inter-visit-interval during the reversal phase, no learning and memory differences were found between the ALLO treated and control group. No conclusions can be drawn about the possible positive effect of prenatal ALLO treatment on the cognitive performance of LBW piglets. However, we also did not find any indication that ALLO had a detrimental effect on cognitive performance when piglets were tested from seven weeks of age.

Clearly more EpR were made during the 1^st^ block of training compared to the last communal block of training (block ten). During the first block, the number of EpR increased with each successive reward in a trial, but no differences between treatment or birth weight groups were seen. During a later stage of training (block ten), the number of errors stayed more or less similar till the 3^rd^ reward is found. This measure reflects that 1) most animals reached a high, but not errorless performance level, and 2) that in particular from the 3^rd^ reward onwards the task becomes more difficult for most of the pigs. The latter could be related to the attention span of the animals or their memory load capacities and is a fact to keep in mind when defining the difficulty level of a learning task. Although attention span is found to be impaired in (v)LBW children [59], this was not confirmed in our LBW piglets by the EpR analysis.

No treatment effects of ALLO were found on the food search strategy, as reflected by the measure CC. Both BW groups were found to apply a partial strategy per specific entry door, but no clear development of a search strategy was seen over blocks, when ‘door’ was not included in the analysis. All CC scores per door clearly differ from the random performance score 1.72, calculated over all training trials of both BW groups [60]. As average CC scores are found to be 2.263 (± SEM 0.139) for the LBW and 2.257 (± SEM 0.131) for the NBW animals, it is clear that the animals adopt a search pattern per entry door, although optimal performance (a score of 4) was never reached. According to Oades [75] a fixed search pattern in the holeboard can be considered as reflecting efficient learning. We did not detect one specific food search strategy if the analysis was run across all entry doors. However, if entry door was considered in the analysis, it became obvious that the search pattern differed per entry door. This shows that most pigs are able to develop a (partial) strategy per entry door. This accounts for both BW groups and strengthens the idea that having more than one entry door increases the difficulty of the task [45].

### Brain, Spleen and Body Weight

The LBW piglets did not show compensatory weight gain and their final body weight was still lower compared to the average weight of NBW animals at slaughter. Lasting effects on body weight are in agreement with previous studies [61], [62] and are also observed in human LBW children [63]. Additionally, a marginal treatment by birth weight interaction effect was found, suggesting that the birth weights of ALLO treated LBW pigs were higher than those of the untreated LBW animals. This effect of ALLO was not found in the NBW pigs. This effect is not reflected by the relative brain and hippocampus weights of the animals. The findings are in agreement with the data from LBW children that remain atypically small during early years and run a larger risk of less than optimal cognitive development [64]. Therefore postnatal growth is an important developmental factor.

Spleen weights (corrected for body weight, as spleen weight increases with body weight [65]) of ALLO treated animals tended to be lower than those of controls. In particular, the relative spleen weights of ALLO treated but NBW animals tended to be lower than those of the other three groups. A characteristic of chronic stress (as we hypothesized to occur during the prenatal period of LBW piglets) is a change of size in stress-related tissues [66]. Long-lasting stress is known to decrease the weight of organs such as the spleen [67]–[69], but this involution of the organ is also found to eventually return to normal after termination of stress [70]. On the contrary, Blanchard et al [66] found spleen weights corrected for body weight to increase in chronically stressed animals, but this may reflect an inflammatory response to wounding as male dominance hierarchies were studied in their experiment. Furthermore, the actual organ weight is largely determined by the actual blood flow and erythrocyte storage into this organ [71]. It would be premature to indicate whether and how stress levels were influenced by ALLO treatment in one of the BW groups based on this marginal finding and considering the discrepancies in literature, but this does indicate a potential avenue for future research.

Neither relative brain nor hippocampus weights were influenced by ALLO treatment in any direction. Both measures are found to be higher in LBW compared to NBW animals, whereas absolute weights were lower. In preterm (v)LBW children, lower brain and hippocampal weights were found [72]. In human children, head circumference (a measure correlating to brain weight) at eight months of age appears to be the best growth parameter for predicting IQ at the age of three years. Adequate compensatory brain growth during the first year of life could prevent much of the negative effects on IQ at three years of age [73]. The brains of the pigs in this study were weighed at 5–5.5 months of age. Because the LBW piglets selected probably suffered from relatively mild IUGR, partial compensatory postnatal brain growth could have taken place.

### Conclusions

The aim of this study was to assess both safety and efficacy of prolonged prenatal oral ALLO treatment in piglets via the sow. Preliminary analysis of the plasma concentrations in sows and their piglets suggested that a daily dose of 15 mg.kg^−1^ results in effective plasma concentration of ALLO in piglets. In contrast to studies with other animal species as well as humans, only relevant ALLO but not OXY levels were measured in the unborn/neonatal piglets and no accumulation of the drug was measured in the sows.

ALLO treatments, even over a slightly longer period, had no adverse effects on farrowing, confirming previous findings in pigs by Boda et al. [31]. These authors applied a dose of 30 mg.kg^−1^ during 4–8 days preceding delivery. In the present study, none of the piglets sampled showed blood gas values indicating that they had suffered from acute birth hypoxia.

The placental circumference was found to correlate with piglet birth weight. ALLO treated piglets seemed to have shorter placentas. As the treated pigs were also found to have taller bodies, placenta length does not seem to be a naturally relevant factor influencing the growth of treated piglets. No interaction effects between treatment and birth weight were found.

An open field test for emotional reactivity at five weeks of age did not reveal any differences between treated and untreated animals. Though, LBW animals were found to vocalize more compared to NBW siblings. Anxiety levels in LBW piglets may be increased, as is found for LBW human children. We therefore suggest this is a shared phenomenon between humans and pigs.

Evaluating the cognitive capacities of ALLO treated piglets in the cognitive holeboard task we could not identify any effects of the ALLO treatment, nor were there any differences between LBW and NBW piglets. These findings contrast with the results of a previous study in which we observed differences in response flexibility between LBW and NBW piglets after switching to a new configuration [22]. This discrepancy might be attributable to the fact that the experimental unit differed between both experiments (affecting statistical power and sensitivity). Also, in the present study we trained animals until a specific (higher) level of performance was reached. However, results clearly indicated that a prolonged prenatal treatment with ALLO can be regarded as safe as no undesirable side effects on cognitive performance were observed.

LBW piglets did not reach the same final body weights as NBW animals, but body weight at 5 to 5.5 months of age showed evidence of postnatal compensatory growth, as did brain and hippocampus. LBW animals treated with ALLO showed the largest postnatal compensatory body weight gain, a positive indication for the chronic prenatal use of ALLO in these animals. Further research should take into account that relative spleen weights tended to be lower in treated NBW animals, although relative brain and hippocampus weights were not influenced by treatment.

We conclude that prolonged prenatal ALLO treatment during the third trimester in sows and their LBW and NBW piglets is safe during pregnancy and delivery, and did not affect the postnatal period. The efficacy of treatment on the cognitive performance of the piglets remains unclear, despite the fact that the plasma-concentrations time curves measured in sows and also the piglets confirmed the diaplacental transfer of ALLO reaching steady state concentrations [74] which are believed to be therapeutically active. Relative brain and hippocampus weights seem to be unaffected by treatment but the final growth of treated LBW pigs appears to be improved compared to the other three groups.

## Supporting Information

Text S1 **This text contains Table S1 that lists all experiments performed.**
(PDF)

Text S2 **This text reports allopurinol (ALLO) and oxypurinol (OXY) plasma levels in sows and their piglets (experiment S2a) and possible short-term effects of chronic allopurinol treatment on birth measures and placental measures (experiment S2b).** The text contains Tables S2.1, S2.2 and S2.3, and Figure S2.1
(PDF)
