# Supplementary material for: Chronic Allopurinol Treatment during the Last Trimester of Pregnancy in Sows: Effects on Low and Normal Birth Weight Offspring
Source: PLoS One. 2014 Jan 22;9(1):e86396. doi: 10.1371/journal.pone.0086396 (PMC3899238; doi:10.1371/journal.pone.0086396)
Supplement: Text S1 — This text contains Table S1 that lists all experiments performed. (PDF) [file pone.0086396.s001.pdf]

Text S1

An overview of the exploratory pharmacokinetic experiments and of the short-term effects of chronic allopurinol treatment during the last trimester of pregnancy (S2a, S2b and S2c, fully reported in Supporting information S2) and of the main study (fully reported in the article) is shown in Table S1.

Table S1. Overview of all experiments performed. *Abbreviations: Exp. = experiment; ALLO = Allopurinol; NBW = normal birth weight; LBW = low birth weight*

Experi-ment	Subjects	Treatment	Pharmacokinetics	Placental measures	Behavior	Organs
**Exp. S2a**	**Two cathe-terized pregnant sows**	PO administra-tion of ALLO on day 99 and 110 of pregnancy; IV administration on day 107 of pregnancy.	Samples of both sows collected on days 99 and 107 for non-compartmental pharmacokinetic analysis; Samples from piglets of one sow on day 110 delivered by Caesarean section. Data of second sow and her piglets were not analyzed due to technical problems			
**Exp. S2b**	**Two cathe-terized pregnant sows**	PO administra-tion of ALLO on day 107 and 113 of pregnancy	Allo- and Oxypurinol measurement once, approx. at expected peak concentration of ALLO in sows and piglets delivered by Caesarean section			
**Exp. S2c**	**Five pregnant sows**	Two sows chronically treated with ALLO during the last 30 days of pregnancy, three untreated control sows		Piglet-matched placentas for determining placenta weight, length, width, circumference, surface, general appearance		
**Main study**	**Twelve pregnant sows in two batches of 6 sows**	Batch 1 and 2: Three sows chronically treated with ALLO during last 30 days of pregnancy, three untreated control sows; selection of LBW and NBW piglets.		Piglet-matched placentas for determining placenta weight, length, width, circumference, surface, general appearance (not restricted to the LBW and NBW piglets selected for behavioral testing); Blood sample from umbilical cord for blood gas analyses	Behavioral testing starting after weaning: open field and novel object test; spatial holeboard, acquisition and reversal	Slaughter at 5 to 5.5 months of age: body weight, weight of brain, hippocampus, spleen
