# Supplementary material for: Chronic Allopurinol Treatment during the Last Trimester of Pregnancy in Sows: Effects on Low and Normal Birth Weight Offspring
Source: PLoS One. 2014 Jan 22;9(1):e86396. doi: 10.1371/journal.pone.0086396 (PMC3899238; doi:10.1371/journal.pone.0086396)
Supplement: Text S2 — This text reports allopurinol (ALLO) and oxypurinol (OXY) plasma levels in sows and their piglets (experiment S2a) and possible short-term effects of chronic allopurinol treatment on birth measures and placental measures (experiment S2b). The text contains Tables S2.1, S2.2 and S2.3, and Figure S2.1 (PDF) [file pone.0086396.s002.pdf]

Text S2

Introduction

In experiments S2a and S2b, bioanalysis of the blood samples to determine allopurinol (ALLO) and oxypurinol (OXY) plasma levels was performed with reversed-phase high-performance liquid chromatography with UV-detection as described in van Kesteren et al. [1], except that the limit of detection was lower for this analysis (0.2 instead of 0.4 µg.ml^{-1} for both ALLO and OXY) than in the study by van Kesteren.

In experiment S2c, the possible short-term effects of chronic allopurinol treatment on birth measures and placental measures were assessed. In addition, these measures (combined with data from the study reported in the article) were used to calculate correlations between placental measures and birth weights.

Material and Methods

Experiment S2a: pharmacokinetics of allopurinol and oxypurinol in sows and piglets

Animals: Two pregnant sows, both a (Terra x Finnish landrace) x Duroc mix, 5th and 9th parity, (see Table S2.1) were used.

Housing: The sows were housed in separate pens but next to each other in an air-conditioned room with an average ambient temperature of 20° C. Light was provided between 07:00 h and 19:00 h. The sows were fed twice daily with a standard pregnant sow diet (de Heus, Ede, The Netherlands), at 07:30 h and 22.00 h, supplemented with silage. They always had ad libitum access to water. The floor of the pens was covered with sawdust and enrichment material (ball or chewing sticks) was provided. Starting from the day of catheterization surgery their health status and surgery wounds were monitored three times a day. The first ALLO administration was scheduled six days after surgery, on day 99 of pregnancy.

Table S2.1. Overview of the sows and piglets used in experiments S2a and S2b. *All animals are a (Terra X Finnish landrace) X Duroc mix. The data collected in sows 1 and 2 on days 99 and 107 were used for non-compartmental pharmacokinetic analysis.*

	Animals	Parity	Weight (kg) at start	Surgery	Piglets used for Allopurinol and Oxypurinol determination (minutes post partum)	Allopurinol treatment (dose, route, day of pregnancy)
Exp. S2a	Sow 1	5	254	catheterized	17 piglets at 225 min. post partum	20 mg.kg^{-1}: PO, day 99; IV, day 107; PO day 110
	Sow 2	9	292	catheterized	Data collected at day 110 were unreliable and excluded from analyses	20 mg.kg^{-1}: PO, day 99; IV, day 107; PO, day 110
Exp. S2b	Sow 3	6	315		5 piglets at 170 min. post partum, 3 piglets at 290 min. postpartum	20 mg.kg^{-1}: PO, day 107; PO, day 113
	Sow 4	5	273		6 piglets, 170 min post partum	20 mg.kg^{-1}: PO, day 107; PO, day 113

Surgical procedures: To be able to take regular blood samples or to administer ALLO without the need to restrain the animal or causing repeated discomfort the sows were fitted with indwelling catheters. Only sows from experiment 1 were subjected to this surgery. The animals were sedated first IM with

azaperone 4mg.kg^{-1} (Stressnill®) and ketamine 2 mg.kg^{-1}. When the animal was calm or asleep, an ear vein was catheterized and additional ketamine (2-4 mg.kg^{-1} IV) and midazolam (0.05-0.1 mg.kg^{-1} IV) was administered. The sow was placed on one side and the surgery areas were cleaned, shaved and disinfected. After preparing the sows for surgery, they were intubated and given isoflurane to maintain the anesthesia.

While the animal was anaesthetized and monitored constantly, a catheter was placed in the left jugular vein. The catheter was tunneled subcutaneously and exteriorized on the back of the sow. Between the shoulder blades a small 'backpack' was adhered to the skin (with medical adhesive and leucoplast tape) to keep the tube and the drip safe and clean. In between blood sampling the tube was constantly filled with a 2 ml heparin solution (50 IE/ml) to prevent blood clotting. The tube was flushed at least once a day and before and after every blood sample; first 3 ml of heparinized physiological salt and some blood was collected from the tube, then the tube was flushed slowly with physiological salt (10 ml) and was finally filled with 2 ml heparinized physiological salt.

After surgery the animals were allowed to recover and were kept warm until they started to eat. To prevent infection around the surgery wounds, antibiotics (benzyl penicillin) were given on the day of surgery and the 2 following days. An analgesic (depomycin) was given for 2 days, starting on the day of surgery.

Drug treatment: Both sows were treated with ALLO orally (20 mg.kg^{-1} body weight) and via intravenous administration (20 mg.kg^{-1} body weight). Treatment days were day 99, 107 and 110 of pregnancy. For oral treatment ALLO tablets (100 mg, Ratiopharm, The Netherlands) were powdered and mixed with standard pregnant sow pellets. To prevent the sows from refusing the food based on an unfamiliar taste, they were habituated to eat these pellets mixed with some water and honey. The experimenter always observed the animal until the entire portion was consumed to confirm complete intake of the intended dose. Animals always consumed their food portion mixed with ALLO within 5-10 minutes.

PO administration of 20 mg.kg^{-1} body weight allopurinol (first measurement): On day 99 of pregnancy, both sows were treated orally with ALLO (20 mg.kg^{-1} body weight). Blood samples were taken as described above. Fifteen minutes before administration a control sample of 4 ml blood was taken via the catheter and stored cold (4°C) in an EDTA tube. Starting 15 minutes after drug administration blood samples (4 ml) were collected every 15 minutes over a period of four hours (16 samples). Thereafter the sampling interval was increased to one hour over a period of seven hours (seven samples), followed by two additional sampling times at 20 and 25 hours p.a. respectively.

IV administration of 20 mg.kg^{-1} body weight allopurinol: On day 107 of pregnancy, both sows were treated with ALLO (100 mg.ml^{-1} in 4 M NaOH solution, sterilized with a 0.2 µm filter, and administered within 24 h after preparation) administered through an intravenous drip. A control blood sample (4 ml) was taken just before the infusion started. The infusions lasted for approximately 20 minutes in sow 1, and 26 minutes in sow 2. After the ALLO infusion the catheter was flushed with 40 ml of NaCl to prevent cross-contamination of the following blood samples. Blood samples were taken at 5, 15, 25, 35, 45, 55, 65, as well as after 125, 185 and 245 minutes (10 samples in total).

PO administration of 20 mg.kg^{-1} body weight (second measurement): On day 110 of pregnancy, both sows were orally treated with ALLO (20 mg.kg^{-1} body weight). Fifteen minutes before administration a control blood sample (4 ml) was taken as described above. 225 Minutes after administration the sow was euthanized with pentobarbital (Euthanimal®, 150 mg.kg^{-1}, Alfasan, The Netherlands) administered through the catheter. Immediately after euthanasia, a Caesarean section was performed to deliver the piglets. Immediately after delivery, a blood sample was taken from every piglet by cardiac puncture. All samples were drawn approximately 225 minutes after treating the sows with

ALLO, because the data from the first measurement showed that at this time point ALLO and OXY reached their peak concentration (C_{max}).

Experiment S2b: pharmacokinetics of allopurinol in sows and piglets

Animals: Two pregnant sows, both a (Terra x Finnish landrace) x Duroc mix, 5th and 6th parity, were used (see Table S2.1).

Housing: The sows were group housed in a conventional housing system for pigs with an average ambient temperature of 20°C. Light was on between 07.00h – 22.00h and the sows were fed twice daily with a standard pregnant sow diet (de Heus, Ede, The Netherlands) at 08.00h and 16.00h in two separate pens. The sows had ad libitum access to water and their health status was monitored every day. The concrete floor was partially covered with sawdust and enrichment materials (ball and chewing sticks) were provided. Animals were given 5ml altrogenest (Regumate®, MSD Animal Health, The Netherlands) daily with their food starting four days prior to the expected farrowing date to prevent early farrowing.

Treatment: Oral ALLO (20 mg.kg^{-1} body weight) administration was as described in experiment 1a. Starting on the afternoon before the day of drug administration, animals were fasted. Sows were ALLO treated twice (day 107 and 113 of pregnancy) with six days between treatments.

PO administration of 20 mg.kg^{-1} body weight: On day 107 both sows were orally treated with ALLO (20 mg.kg^{-1} body weight) and one blood sample (4 ml) was taken from the external jugular vein 170 minutes after ALLO administration. ALLO and OXY plasma levels were determined to assure that these corresponded to the expectations based on experiment 1a. As this was the case, the experiment was continued on day 113 of pregnancy. Both sows were orally treated with ALLO (20 mg.kg^{-1} body weight). Fifteen minutes before administration a control blood sample (4 ml) was taken as described above. 170 minutes after administration the sow was euthanized with an IV injection with pentobarbital (Euthanimal®, 150 mg.kg^{-1}, Alfasan, The Netherlands). Immediately after euthanasia, a caesarean section was performed to deliver the piglets. Through a cardiac puncture a blood sample was withdrawn from every piglet before the animal was euthanized.

The samples were drawn 170 minutes after ALLO treatment. This was done because the data gathered in exp. 1a showed OXY levels after ALLO administration to be very low in all piglets (and therefore therapeutically marginal). 170 Minutes was the average C_{max} of ALLO in the sows from experiment 1a and therefore at 170 minutes we expected to measure around the ALLO plasma level peak of the piglets too. Additionally three piglets were sampled at t=290 min.

Pharmacokinetics of allopurinol: Plasma ALLO and OXY analysis in the sows after PO and IV administration was performed in a non-compartmental model. Fetal plasma concentrations were not analyzed statistically because they were measured at only one time-point after administration.

Experiment S2c: chronic allopurinol administration in pregnant sows

Animals: Five multiparous pregnant sows, a (Terra x Finnish landrace) x Duroc mix, were used (for details see Table S2.2).

Housing: The sows were housed in a group housing system with automatic feeders, straw bedding and *ad libitum* access to water. The sows could freely enter an outside area where silage was

provided. The ambient inside temperature ranged between 15 and 25°C and light was provided between 07:00 h and 22:00 h. Except for 1 kg of pellets mixed with ALLO, the daily food ration (standard pregnant sow pellets, de Heus, Ede, The Netherlands) was distributed via an automatic feeder.

Table S2.2. Overview of the sows and piglets used in experiment S2c*. All animals are a (Terra x Finnish landrace) x Duroc mix.*

Animals	Treatment	Parities	Litter size	Average litter weight (g)	Treatment
Sow 1	Allopurino	9	13	1720.83	15 mg.kg^{-1} PO (once daily)
Sow 2	Allopurinol	7	15	1617.47	15 mg.kg^{-1} PO (once daily)
Sow 3	Control	7	15	1308.33	none
Sow 4	Control	7	14	1040.00	none
Sow 5	Control	6	16	1000.00	none

Drug treatment: Based on the results of our exploratory pharmacokinetics studies (see Exp. S2a and S2b in Supporting Information S2) two sows were treated with allopurinol (15 mg.kg^{-1} b.w.) for 30 days [± 2 days depending on the actual farrowing date, starting at day 86 (+ 1-3 days) of pregnancy] and three untreated sows served as controls (see Table S2.2). ALLO tablets (300 mg, Ratiopharm, The Netherlands) were powdered and mixed with 1 kg of pellets, some honey and water. Animals were observed until all the food was consumed. Sows were weighed weekly to adapt the dose corresponding to their weight gain or loss. As the Caesarean sections were planned on day 114 of pregnancy, all sows were given 5 ml Altrogenest (Regumate®, MSD Animal Health, The Netherlands) daily with their morning food starting four days prior to the expected farrowing date to prevent early farrowing.

Procedures around delivery

Sows: Directly after restraining the sow, unconsciousness was reached by captive bolt stunning. Piglets were delivered via a Caesarean section and the sow was bled. When a piglet was delivered the umbilical cord was tagged twice with coded umbilical clamps and cut in between the two clamps. This enabled linking a placenta to a specific piglet.

Piglets: Piglets were brought to a recovery room where they were dried and examined. Mucus was removed from the mouth and snout and the piglets were weighed, measured (length from nose to tail base and nose to end of the skull) and ear tagged. The piglets were housed in a small pen which was partially covered and heated with heat lamps and a heat mat (ambient temperature 32°C). No further measurements were performed on the piglets.

Placental measures: All placentas that could be gathered from a sow were stored (4°C) and examined within one week to look at any possible adverse effects of ALLO treatment on placental development. Measures included: placenta length (measured along the inside of the placenta from one end to the other); placenta width (measured along the base of the placenta at the broadest point); and placenta circumference (measured by placing a piece of string exactly around the edges of the placenta). All placentas were weighed (10 g accuracy, Breuer Weegtechniek JB-800, Boxtel, The Netherlands). Scaled pictures were taken from above to calculate the surface area using PDF-Xchange Viewer 2.5. Only placentas that were mainly undamaged and could be unfolded were measured.

Results

Experiment S2a: pharmacokinetics in catheterized sows

Sows: A non-compartmental analysis was performed on the data of the (IV) infusion of an ALLO dose of 20 mg.kg^{-1} body weight and a PO administration of 20 mg.kg^{-1} body weight The results are summarized in Table S2.3.

Table S2.3. Pharmacokinetic parameters of plasma allopurinol (ALLO) and oxypurinol (OXY) in sows 1 and 2, non-compartmental analysis.

Parameters	Units	Dose and route of administration			
		20 mg.kg^{-1} PO (ALLO)		*20 mg.kg^{-1} IV (ALLO)*	
		Sow 1	*Sow 2*	*Sow 1*	*Sow 2*
		Mean	*Mean*	*Mean*	*Mean*
C_{max}	µg.ml^{-1}	6.00	6.98	19.85	27.77
T_{max}	hr	4.00	2.75	0.60	0.52
λ_z	1.hr^{-1}	0.46	2.60	0.65	0.70
HL λ_z	hr	1.50	0.37	1.07	0.99
AUC_{last}	hr.µg.ml^{-1}	22.83	21.46	29.72	35.07
$AUMC_{last}$	hr.hr.µg.ml^{-1}	72.43	62.80	43.38	48.65
MRT_{INF}	hr	3.20	2.93	1.57	1.60
		20 mg.kg^{-1} PO (OXY)		*20 mg.kg^{-1} IV (OXY)*	
C_{max}	µg.ml^{-1}	4.51	5.17	6.64	8.17
T_{max}	hr	4.50	4.00	0.60	0.52
λ_z	1.hr^{-1}	2.67	1.21	0.29	0.28
HL λ_z	hr	0.26	0.57	2.37	2.43
AUC_{last}	hr.µg.ml^{-1}	17.50	20.22	17.41	22.25
$AUMC_{last}$	hr.hr.µg	63l31	71.22	32.93	43.56
MRT_{INF}	hr	3.66	3.61	3.65	3.77
AUC_{oxy}/AUC_{allo}		0.77	0.94	0.59	0.63

C_{max} = maximal plasma concentration; T_{max} = time to reach C_{max}; λ_z = elimination constant; HL λ_z = elimination half-life; AUC_{last} = Area Under the plasma concentration-time Curve from time zero to time of last measurable concentration; $AUCM_{last}$ = Area under the first moment of the plasma concentration-time curve from time zero to time of last measurable concentration; MRT_{INF} = Mean Residence Time to infinity; AUC_{oxy}/AUC_{allo} = Area Under the Plasma concentration time Curve OXY / Area Under the Plasma concentration time Curve ALLO. PO measurements at 99, and IV measurements at 107 days of gestation.

In the two sows the mean C_{max} for respectively PO and IV dosing of ALLO 20 mg.kg^{-1} was 6.49 and 23.81 µg.ml^{-1} for ALLO and 4.84 and 7.41 µg.ml^{-1} for OXY. After oral dosing ALLO and OXY plasma levels were below the limit of detection (LOD) at >25h (ALLO) and >25h (OXY) for sow 1, and 25h (ALLO) and >25h (OXY) for sow 2. After IV dosing, in both sows plasma ALLO and OXY levels were still detectable >4 hours after administration.

Piglets: A complete set of plasma concentrations is only available from the piglets of sow 1, from which all 17 piglets were sampled. The average ALLO and OXY plasma levels were 4.10 µg.ml^{-1} and 0.45 µg.ml^{-1} respectively, 225 minutes after oral administration of ALLO to the sow (see Fig. S2.1, panel A). The sow plasma level samples at 225 min were 7.69 µg.ml^{-1} (ALLO) and 5.56 µg.ml^{-1} (OXY).

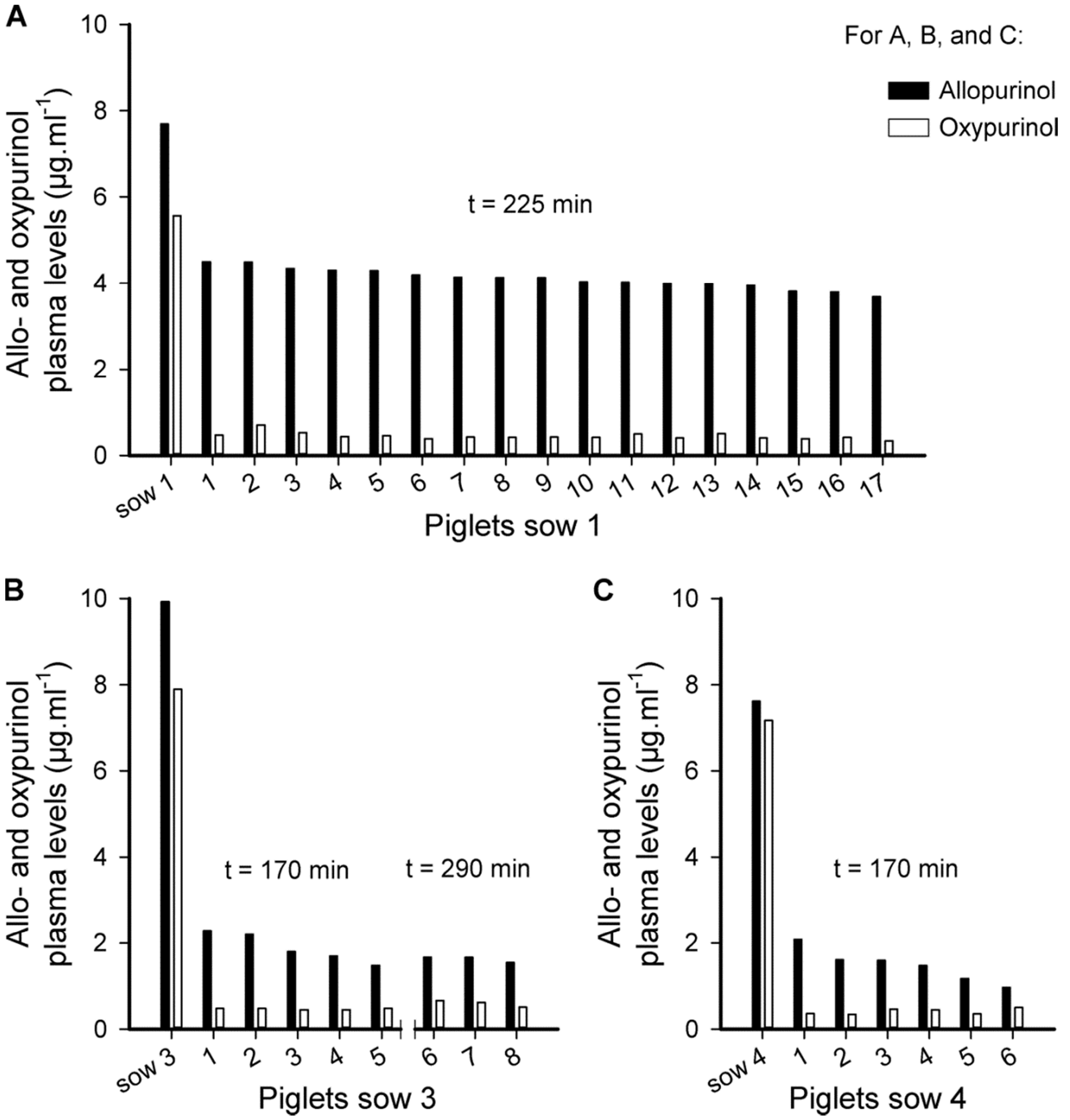

***Figure S2.1**. **Allopurinol and oxypurinol plasma levels** (µg.ml^{-1})**.** The plasma levels were measured in three different sows and their piglets (sow 1: panel A, sow 3: panel B, sow 4: panel C; for details see Table S2.1). The time points of sampling post partum are indicated in the figure.*

Experiment S2b: pharmacokinetics in sows and piglets

As shown in Figure S2.1, panel B and C, five piglets from sow 3 and six piglets from sow 4 were sampled at 170 minutes after ALLO administration. In addition, 3 piglets of sow 3 were sampled at 290 min *post partum*. Average ALLO plasma levels were found to be 1.80 µg.ml^{-1} and 1.49 µg.ml^{-1} respectively in the piglets of sow 3 and 4. Average OXY plasma levels of 0.53 µg.ml^{-1} (piglets of sow 3) and 0.42 µg.ml^{-1} (piglets of sow 4) were found. The sows were sampled at the same time point as their piglets.

Experiment S2c: chronic allopurinol administration in pregnant sows

The appearance of all piglets and of their placentas was normal, suggesting that the chronic treatment with allopurinol during the third trimester of pregnancy had no adverse effects.

The birth weight and placental measures of the piglets in this experiment were analyzed together with the corresponding data from the main study. Results are summarized in Table 2. Correlations between piglet birth weight and all traceable placentas are depicted in Table 3. Note that the mean of the measures per sow was used, i.e. the maximum number of values was 17 (12 sows from the main study, and 5 sows from exp. S2c).

Discussion

Experiments S2a and S2b confirmed an earlier study by van Dijk and colleagues that allopurinol readily crosses the placental barrier [2]. The present results were used to establish the dosing regimen for the long-term application of ALLO to pregnant sows. Considering a desirable plasma concentration in the piglets of at least 2 µg.ml^{-1} ALLO, a simulation of the kinetic parameters obtained from sow 1 and her piglets suggested that a minimum oral dose of 14 mg.kg^{-1} body weight would be required to achieve steady state conditions in piglets and to exclude drug accumulation in the parent animal. Experiment S2c supports that notion that chronic allopurinol treatment was safe, as neither the piglets nor the placentas macroscopically showed any abnormalities. Hence it was decided to apply an oral dose of 15 mg.kg^{-1} body weight in the main study.

References

1. van Kesteren C, Benders JNL, Groenendaal F, van Bel F, Ververs T, et al. (2006) Population pharmacokinetics of allopurinol in full-term neonates with perinatal asphyxia. Ther Drug Monit 28: 339.

2. van Dijk AJ, Parvizi N, Taverne MAM, Fink-Gremmels J (2008) Placental transfer and pharmacokinetics of allopurinol in late pregnant sows and their fetuses. J Vet Pharmacol Ther 31: 489-495.
